# Causal relationship between body mass index and risk of juvenile idiopathic arthritis: A 2-sample Mendelian randomization study

**DOI:** 10.1097/MD.0000000000047446

**Published:** 2026-01-30

**Authors:** Renkun Huang, Guohua Jiang, Jiehua Lu, Yuchang Gui, Bingtao He, Chaoquan Yang, Jianwen Xu

**Affiliations:** a Department of Rehabilitation Medicine, The First Affiliated Hospital of Guangxi Medical University, Nanning City, Guangxi, China; b Ruikang Hospital, Affiliated to Guangxi University of Chinese Medicine, Nanning City, Guangxi, China; c Department of Bone and Joint Surgery, The First Affiliated Hospital of Guangxi Medical University, Nanning City, Guangxi, China.

**Keywords:** body mass index, genetic association, juvenile idiopathic arthritis, Mendelian randomization

## Abstract

The association between body mass index (BMI) and juvenile idiopathic arthritis (JIA) has been suggested, but the causal relationship remains unclear. Mendelian randomization (MR) offers a tool to address this causal question using genetic data. This study aims to investigate the causal relationship between BMI and JIA, providing genetic evidence to inform clinical prevention and treatment strategies. A total of 35 BMI-related single nucleotide polymorphisms were included as instrumental variables. Inverse variance weighting analysis revealed a significant positive association between increased BMI and higher JIA risk (odds ratio = 1.000388, 95% confidence interval: 1.000001–1.000776, *P* = .0494). No significant association was found in MR-Egger (*P *= .05154), weighted median estimator (*P* = .1959), or weighted models (*P* = .3698). The MR-Egger regression intercept was 0.019 (*P* = .453), indicating no significant pleiotropy, and no bias was detected in the leave-one-out sensitivity analysis or funnel plots. This study provides genetic evidence supporting a weak positive causal relationship between increased BMI and a higher risk of JIA. However, the clinical significance of this association is limited. Summary-level data were obtained from public genome-wide association studies. Single nucleotide polymorphisms associated with BMI (*P *< 5 × 10^−8^, *R*^2^ < 0.001) were selected as instrumental variables. The BMI dataset included 99,998 participants, and the JIA dataset included 15,872 participants. The primary analysis methods were inverse variance weighting, MR-Egger regression, and weighted median estimator, with additional weighted models. Sensitivity analysis was performed using the leave-one-out method, and pleiotropy was assessed using MR-Egger regression intercept. Heterogeneity was evaluated using Cochran *Q* test and funnel plots, and scatter plots were used to assess consistency in effect directions.

## 1. Introduction

Juvenile idiopathic arthritis (JIA) is not a single disease but a cluster of chronic autoimmune inflammatory conditions, defined by onset before 16 years of age, duration of at least 6 weeks, and involvement of 1 or more joints; its exact etiology remains unclear.^[1]^ As one of the most prevalent chronic inflammatory diseases in childhood, JIA has a global prevalence of approximately 1 in 1000. Although its pathogenesis has not been fully elucidated, it is widely accepted that the interplay between genetic susceptibility and environmental factors plays a pivotal role in disease initiation, which can lead to systemic symptoms and joint tissue damage.^[2,3]^

Body mass index (BMI) is an internationally recognized key indicator for evaluating an individual’s weight status and health level. A growing body of observational studies has suggested that higher BMI may be associated with an increased risk of JIA onset.^[4,5]^ However, observational studies are inherently susceptible to confounding factors and reverse causality, which hinder the accurate determination of whether a causal relationship exists between BMI and JIA. Therefore, additional methodological approaches are required to further validate this potential association.

Mendelian randomization (MR) is a causal inference method based on genetic variations. This approach utilizes genetic variants as instrumental variables (IVs) to analyze the causal relationship between an exposure and a disease outcome, thereby effectively minimizing the interference of confounding factors on study results.^[6]^ Furthermore, since genetic variants representing the exposure are not influenced by the occurrence or progression of the disease, MR studies can also reduce the likelihood of reverse causality.^[7]^

Based on summary-level data of single nucleotide polymorphisms (SNPs) from publicly available genome-wide association studies (GWAS), the present study employed a 2-sample MR design to investigate the causal relationship between BMI and JIA. The ultimate goal was to provide more robust evidence for understanding the potential link between these 2 factors.

## 2. Methods

### 2.1. Study design

Causal association analysis was performed with JIA as the outcome variable and BMI as the exposure factor. The analysis was conducted using the TwoSampleMR package in R software (version 4.5.1; R Foundation for Statistical Computing, Vienna, Austria). To verify the robustness and reliability of the results, Cochran *Q* heterogeneity test, multiple testing, and sensitivity analysis were employed.

### 2.2. Inclusion criteria

First, criteria for defining overweight and obesity based on BMI: BMI was calculated by measuring participants’ height and weight using the formula: BMI = weight [kg]/height^2^ [m^2^]. In accordance with the BMI classification criteria of the World Health Organization, a BMI ≥ 25 kg/m^2^ is defined as overweight, and a BMI ≥ 30 kg/m^2^ is defined as obesity. Second, diagnostic criteria for JIA: the diagnosis was determined with reference to the relevant criteria in the Chinese Expert Consensus on Diagnosis and Treatment of Systemic Juvenile Idiopathic Arthritis (2019 Version).^[8]^

### 2.3. Data sources

The summary-level data used in this study were obtained from the UK Biobank GWAS database (https://gwas.mrcieu.ac.uk/), utilizing publicly available GWAS meta-analysis statistics. Specifically, the BMI dataset (serving as the exposure variable) was derived from a GWAS study published in 2022, while the JIA dataset (serving as the outcome variable) was obtained from GWAS published results in 2013; both datasets were of European ancestry. The 2-sample MR analysis satisfied the independence assumption, and BMI-related data in linkage disequilibrium (LD) with JIA were excluded. All data were retrieved from public databases, and the original studies had obtained informed consent from participants; therefore, no additional ethical approval was required for the present study (Table 1).

**Table 1:** Summary information of GWAS data.

Name	ID	Sample size	SNPs	Consortium	Year	Population
BMI	ieu-b-4816	99998	7191606	Within family GWAS consortium	2022	European
JIA	ebi-a-GCST005528	15872	103767	NA	2013	European

BMI = body mass index, GWAS = genome-wide association studies, ID = identity document, JIA = juvenile idiopathic arthritis, SNPs = single nucleotide polymorphisms.

### 2.4. IVs

To meet the assumptions of MR analysis and avoid analytical bias caused by strong LD between SNPs, IVs were selected through the following steps:

1. The “harmonize_data” function in the TwoSampleMR package was used to standardize the allele directions of exposure and outcome, and SNPs reaching the genome-wide significance level (*P* < 5 × 10^−8^) were screened from the BMI dataset.
2. The threshold for LD was set to *R*^2^ < 0.001, and the threshold for physical distance was set to 10,000 kilobase pairs (kb).
3. Weak IVs were excluded via *F*-test to reduce result bias: SNPs with an *F*-statistic > 10 were included as IVs, while those with an *F*-statistic < 10 were considered weak IVs and excluded.^[9]^

### 2.5. MR analysis

Five estimation methods were employed in the 2-sample MR analysis, with weighted median estimator (WME), inverse variance weighting (IVW), and MR-Egger regression^[10]^ serving as the primary approaches. Studies have shown that the standard error of MR-Egger estimates is generally larger than that of IVW, and its statistical power is inferior to IVW;^[11]^ when there are discrepancies in results across different methods, IVW is typically considered the primary reference.

The key differences among the 3 primary methods are as follows: MR-Egger regression accounts for pleiotropy reflected by the intercept term through intercept analysis; IVW assumes that all SNPs are valid IVs (i.e., satisfying the 3 basic assumptions of MR) and obtains unbiased estimates by calculating a weighted average, making it the most powerful effect estimation method in MR analysis;^[12]^ WME represents the median of the weighted empirical density function of ratio estimates, which can be used to assess causal relationships when the proportion of valid IVs is close to 50%. An intercept close to 0 indicates the absence of pleiotropy.

In the analysis, odds ratio (OR), 95% confidence interval (CI), and *P*-value were used to determine the significance of effects: a *P*-value > .05 indicates no statistical significance. Meanwhile, weighted models were adopted as auxiliary analytical methods to comprehensively evaluate the causal relationship between BMI and JIA.

### 2.6. Sensitivity analysis

Heterogeneity test was used to evaluate differences among IVs. Cochran *Q* test was employed to analyze heterogeneity among SNPs, with a *P*-value < .05 indicating significant heterogeneity. The leave-one-out method was applied to assess the stability of the association between exposure and outcome: this involved removing 1 SNP at a time and repeating the analysis to determine whether the results of the MR model constructed by the remaining SNPs deviated significantly from the overall results. Egger-intercept test was used to evaluate the presence of horizontal pleiotropy, primarily through MR-Egger intercept analysis. A *P*-value > .05 suggested no significant horizontal pleiotropy; if pleiotropy existed, the MR assumptions would be violated.^[13]^ Meanwhile, scatter plots were generated to assess the effects of IVs on exposure and outcome; forest plots were used to present the strength of the association between exposure and outcome; and funnel plots were employed to evaluate data heterogeneity.

## 3. Results

### 3.1. SNP data associated with the relationship

For the 2-sample MR analysis with BMI as the exposure and JIA as the outcome, data were harmonized using the “harmonize_data” function in the TwoSampleMR package. After excluding IVs in LD, 44 BMI-related SNPs were initially selected as candidate IVs. Among these, 9 SNPs (rs10099330, rs10144067, rs10169594, rs10182416, rs10417386, rs1048932, rs1064213, rs10742752, and rs10760277) were palindromic sequences and identified as outliers via MR-SNIO test, thus being excluded. Ultimately, 35 SNPs were included (*P *< 5 × 10^−8^, *R*^2^ < 0.001), with all *F*-statistics > 10. This indicates that the present study is less likely to be biased by weak IVs, and the statistical power is reliable (Table 2).

**Table 2:** Basic information table of SNPs associated with exposure factors and *F*-value.

SNP	Chr	EAF	*P*_val	β	SE	*R* ^2^	*F*
rs10055107	5	0.127057	4.07E-08	-0.1464	0.0267	6.49294E-05	30.06476093
rs10146690	14	0.215875	2.28E-11	0.1536	0.023	9.63151E-05	44.59897559
rs11030104	11	0.203438	1.01E-11	-0.1575	0.0231	0.000100393	46.4874025
rs12142020	1	0.402414	8.69E-11	0.1278	0.0197	9.08865E-05	42.08500464
rs12423420	12	0.731345	4.58E-08	0.1194	0.0218	6.47854E-05	29.99810289
rs13130484	4	0.433636	1.34E-26	0.2037	0.0191	0.000245594	113.7400586
rs1317867	17	0.511922	3.15E-08	-0.1087	0.0196	6.64244E-05	30.75707768
rs1402807	1	0.435395	1.45E-09	-0.1149	0.019	7.89784E-05	36.57050685
rs2140664	10	0.159815	1.81E-11	0.1786	0.0266	9.7357E-05	45.08143792
rs28590228	19	0.683043	2.86E-12	0.1421	0.0203	0.000105818	48.99978834
rs34045288	6	0.334449	2.53E-11	0.1317	0.0197	9.65177E-05	44.69276477
rs4353774	3	0.608446	4.16E-09	0.112	0.0191	7.42586E-05	34.38487381
rs4670454	2	0.340261	1.12E-10	-0.1258	0.0195	8.98799E-05	41.61886033
rs59778458	3	0.69767	3.82E-08	-0.112	0.0204	6.50964E-05	30.14212278
rs633715	1	0.21032	4.18E-23	0.2227	0.0225	0.00021154	97.96558177
rs6419385	12	0.184807	1.42E-09	-0.1463	0.0242	7.89284E-05	36.54736279
rs6722320	2	0.207758	2.44E-08	0.1322	0.0237	6.71965E-05	31.11460861
rs6735049	2	0.828354	1.04E-35	0.3053	0.0245	0.000335263	155.281445
rs6752378	2	0.486246	4.61E-12	0.1281	0.0185	0.000103543	47.94606024
rs67871383	11	0.317628	3.77E-08	0.1107	0.0201	6.55065E-05	30.33201422
rs7132908	12	0.384455	2.15E-18	0.1702	0.0195	0.000164508	76.18123569
rs7161194	14	0.663943	4.5E-10	-0.1374	0.022	8.42367E-05	39.00553399
rs7180312	15	0.452327	1.63E-12	-0.1332	0.0189	0.000107262	49.66871969
rs7187776	16	0.402134	2.09E-14	0.1475	0.0193	0.000126132	58.40735596
rs729153	3	0.595402	1.71E-08	0.1058	0.0188	6.83968E-05	31.67041548
rs7571826	2	0.330166	1.64E-11	0.1367	0.0203	9.79292E-05	45.34642743
rs7591382	2	0.426703	1.77E-11	-0.1258	0.0187	9.77339E-05	45.25600286
rs8050136	16	0.395102	4.53E-83	0.3688	0.0191	0.000804591	372.832029
rs8064502	17	0.436201	1.59E-09	-0.1133	0.0188	7.84368E-05	36.31969938
rs8089364	18	0.265807	5.2E-33	0.2526	0.0211	0.00030944	143.3177251
rs879620	16	0.613209	3.42E-08	0.1076	0.0195	6.57562E-05	30.44762653
rs943005	6	0.169662	3.39E-15	0.1939	0.0246	0.000134164	62.12744992
rs9568868	13	0.129236	2.01E-08	0.157	0.028	6.7899E-05	31.43991521
rs9630985	2	0.665892	1.87E-09	0.1218	0.0203	7.77461E-05	35.9998445
rs9788550	14	0.247482	8.96E-15	-0.1611	0.0208	0.000129544	59.98774476

Chr = chromosome, EAF = effect allele frequency, *P*_val = *P*-value, SE = standard error, SNPs = single nucleotide polymorphisms.

### 3.2. MR analysis of the relationship between BMI and JIA

**Figure 1.**
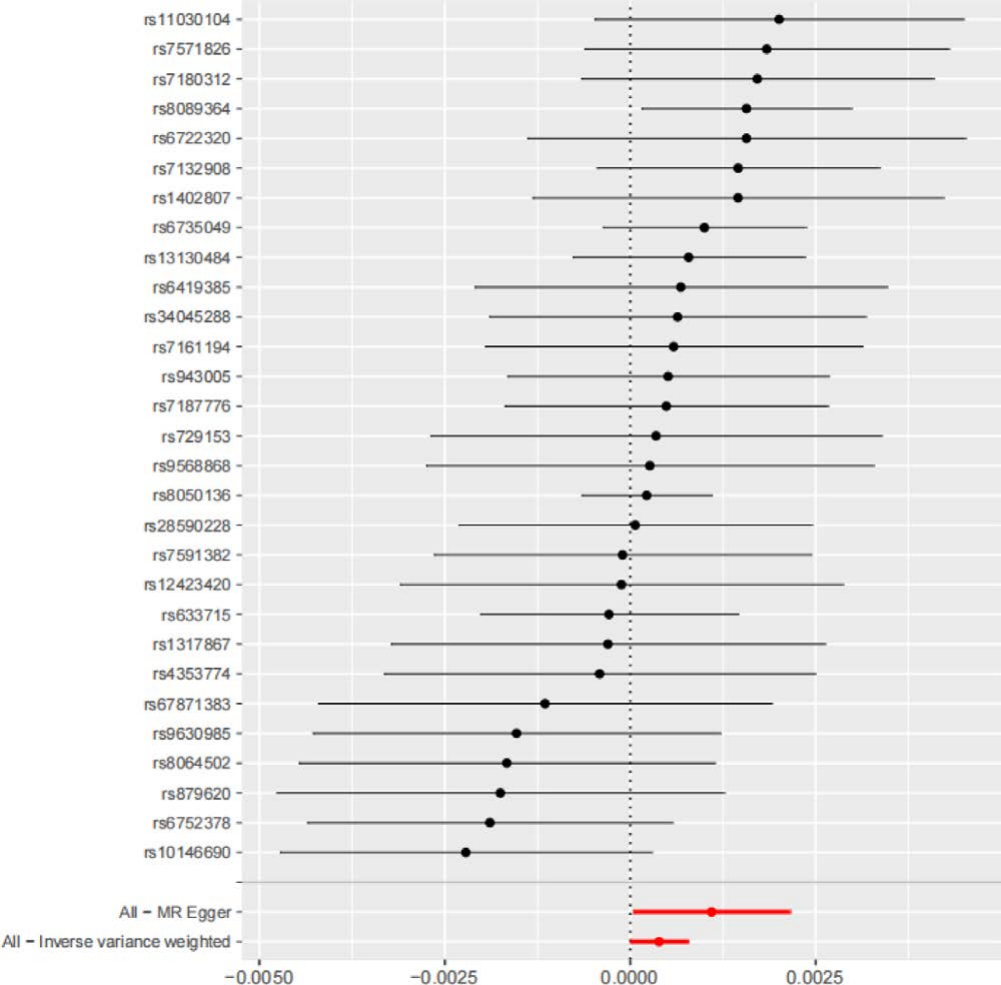
Forest plot of risk relationship.

**Figure 2.**
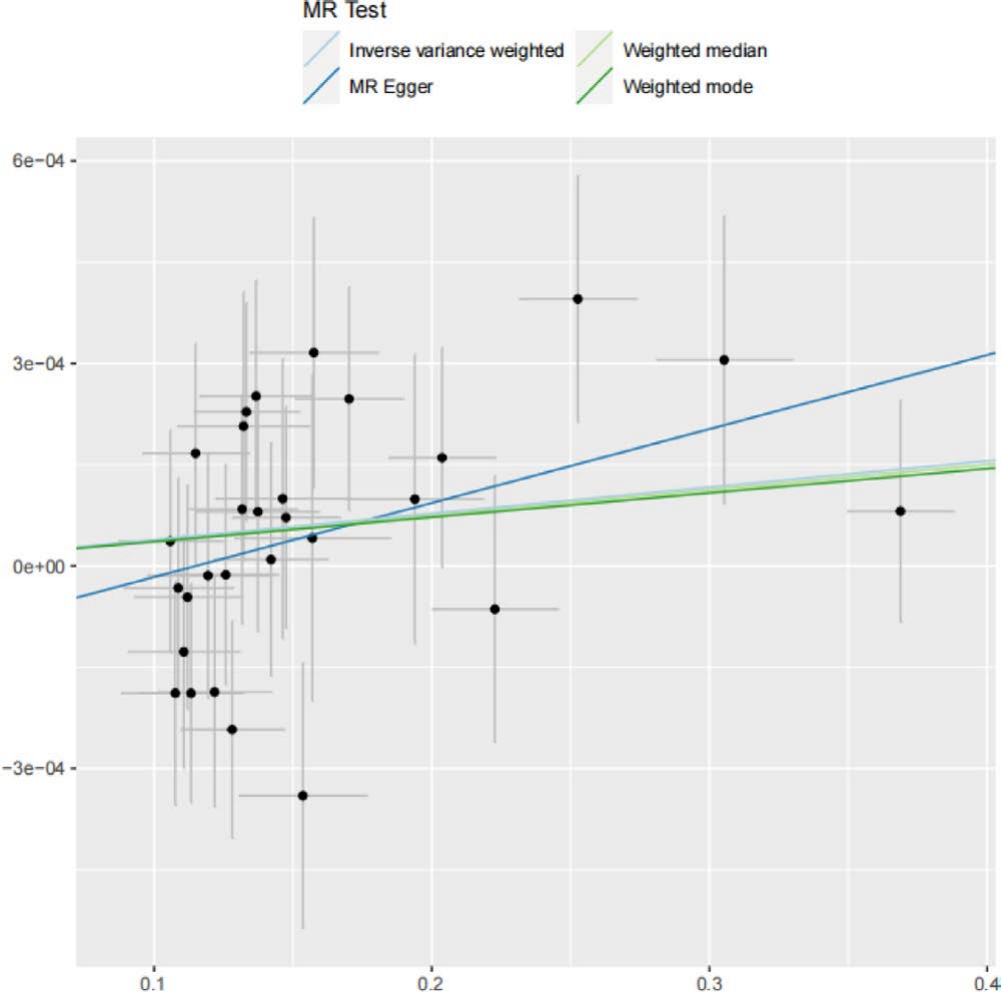
Scatter plot of risk correlation obtained from analysis.

Causal association analysis was performed using the IVW, MR-Egger regression, WME, and weighted models within the TwoSampleMR package. The results were as follows:

For the IVW method, the effect size was calculated as OR = 1.000388 (95% CI: 1.000001–1.000776, *P* = .0494, significance level α = 0.05), indicating that increased BMI is associated with an elevated risk of JIA onset. In contrast, the WME (OR = 1.000375, 95% CI: 0.999807–1.000944, *P* = .1959 > 0.05), MR-Egger regression (OR = 1.001097, 95% CI: 1.000041–1.002153, *P* = .05154 > .05), and weighted models (OR = 1.00036, 95% CI: 0.999586–1.001135, *P* = .3698 > .05) showed no statistical significance. Given the statistical significance of the IVW results, the OR values of all other analytical methods were > 1, and the directions of allelic effect sizes (β) were consistent. These findings collectively suggest a causal association between the exposure (BMI) and the outcome (JIA) (Table 3). The forest plot corresponding to the results showed that the red line for “All-IVW” was entirely on the right side of the null value, further supporting the existence of a causal effect between BMI and JIA under the IVW method (Fig. 1). In the scatter plot of the MR analysis, the *x*-axis represented the effect of SNPs on the exposure (BMI), and the *y*-axis represented the effect of SNPs on the outcome (JIA); the colored lines represented the MR fitting results. The overall slopes of the lines from different algorithms showed an upward trend with slopes > 0, indicating that the exposure (BMI) is an adverse factor for the outcome (JIA), that is, there is a positive correlation between the 2 (Fig. 2).

**Table 3:** Results obtained from the analysis.

Method	β	SE	OR	95% CI	*P*_val
MR-Egger	0.001096	0.0005381	1.001097	1.000041–1.002153	.05154
WME	0.0003751	0.00029	1.000375	0.999807–1.000944	.1959
IVW	0.0003882	0.0003882	0.0001975	1.000001–1.000776	.0494
Weighted mode	0.00036	0.00036	0.0003949	0.999586–1.001135	.3698

CI = confidence interval, IVW = inverse variance weighting, MR = Mendelian randomization, odds ratio = OR, *P*_val = *P*-value, SE = standard error, WME = weighted median estimator.

### 3.3. Reliability analysis

#### 3.3.1. Pleiotropy test

In the Egger-intercept test, the MR-Egger regression intercept was 0.019 with a standard error of 8.6 × 10^−5^ (*P* = .453), indicating the absence of horizontal pleiotropy. Similarly, the MR-PRESSO global test of horizontal pleiotropy was not significant (*P *= .79), further supporting the validity of the instruments.

#### 3.3.2. Heterogeneity test

**Figure 3.**
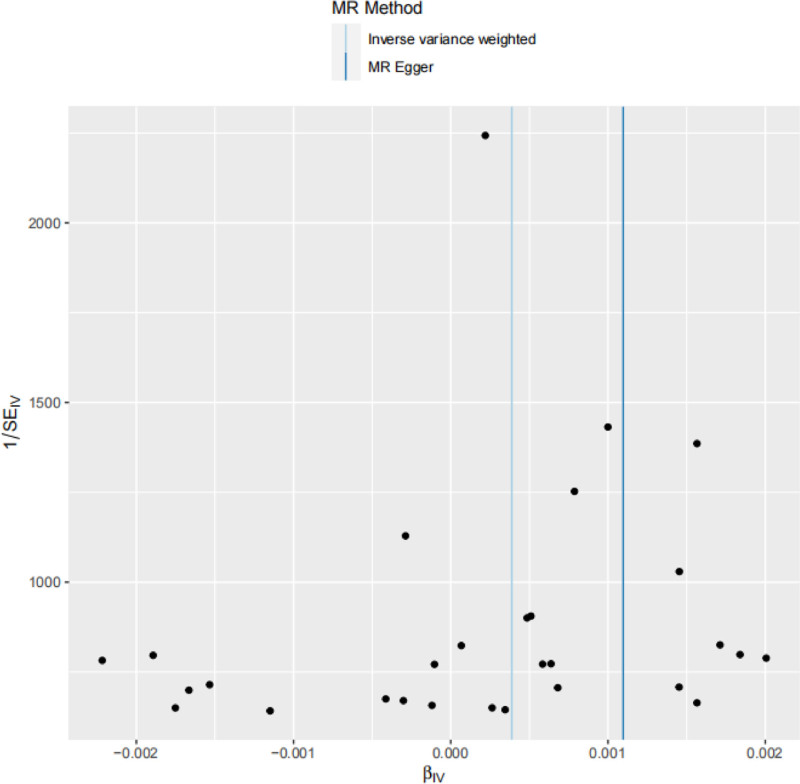
Analysis of the funnel plot for heterogeneity test.

Results of the Cochran *Q* test showed: for MR-Egger, *Q* = 24.21, *P* = .6189, and *I*^2^ = 0.115242; for IVW, *Q* = 26.21, *P *= .5617, and *I*^2^ = 0.068295. Although these results suggested the presence of heterogeneity in the causal relationship between BMI and JIA, this heterogeneity may be attributed to insufficient statistical power (Table 4). All participants included in this study were of European ancestry, and the *I*^2^ values for both methods were < 31%, indicating a low degree of heterogeneity. Previous studies have demonstrated that MR-Egger exhibits good usability and robustness in heterogeneity testing, allowing a certain degree of heterogeneity without affecting result interpretation.^[14]^ Visualization of the results further indicated a low likelihood of bias (Fig. 3).

**Table 4:** Heterogeneity test.

Method	*Q*	*Q*_df	*I* ^2^	*Q*_val
MR-Egger	26.21	28	0.068295	0.6189
IVW	24.21	27	0.115242	0.5617

IVW = inverse variance weighting, MR = Mendelian randomization, *Q*_df = *Q* degrees of freedom, *Q*_val = *Q* value, WME = weighted median estimator.

#### 3.3.3. Sensitivity analysis

**Figure 4.**
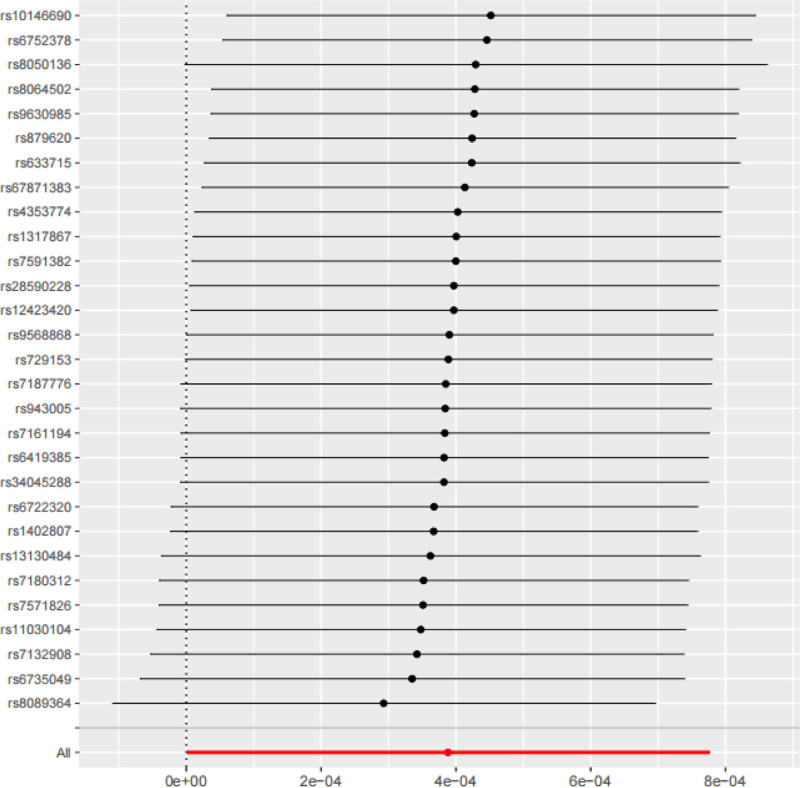
Obtained effect forest plot of the “leave-one-out method” in sensitivity analysis.

The leave-one-out method was used to perform sensitivity analysis on the IVW results, which were also visualized. After removing 1 SNP at a time and repeating the MR analysis, the results showed that all effect sizes were close to the red dot representing the total effect in the figure. No significant bias was observed, and no abnormally significant effect sizes were detected: indicating that the study results were largely unaffected by heterogeneity (Fig. 4).

## 4. Discussion

This study provided evidence supporting the causal association between BMI and JIA via 2-sample MR analysis, further strengthening the causal link between the 2. The results showed that increased BMI may elevate the risk of JIA onset, which is consistent with the conclusions of most observational studies.

Methodologically, a rigorous IV screening process was conducted initially, and 35 BMI-related SNPs were finally included (*P *< 5 × 10^−8^, LD threshold *R*^2^ < 0.001). All included SNPs had an *F*-statistic > 10, fully satisfying the 3 core assumptions of MR analysis and effectively avoiding biases that might be caused by weak IVs. Given the overlap between exposure and outcome among the 35 selected SNPs, 5 estimation methods were used for MR analysis to reduce potential false positives and biases in causal inference: with WME, IVW, and MR-Egger regression as the primary methods. When discrepancies existed between the results of different methods, IVW was taken as the primary reference. However, the MR-Egger and WME methods did not yield statistically significant associations, indicating that the evidence for causality is sensitive to the analytical method used. The final results confirmed that increased BMI raises the risk of JIA: specifically, IVW analysis showed that individuals with higher BMI had a 1.0003-fold increased risk of JIA compared to those with normal BMI. This suggests that scientific management of BMI may provide important references for exploring the etiology of JIA, formulating prevention strategies, optimizing clinical diagnosis, and improving prognosis. Notably, the causal effect size estimated in this study is extremely small and likely of no clinical significance. For instance, a 1 standard deviation increase in BMI corresponded to an OR of only ~1.0017 (approximately + 0.17% odds) for JIA, which is essentially negligible. In contrast, a known strong genetic risk factor such as HLA-B27 confers an odds ratio greater than 10 for JIA, highlighting that the influence of BMI is weaker by orders of magnitude.^[15]^

It should be noted that BMI, as an anthropometric indicator, cannot fully reflect an individual’s health status, actual activity level, or body fat distribution characteristics. For example, adolescents may have a normal BMI, but they still face potential risks of JIA due to immature joint structures during the growth and development stage and high sensitivity of the immune system to inflammatory stimuli.^[16,17]^ Additionally, the investigation of the causal relationship between BMI and JIA in this study is still a preliminary statistical analysis targeting a specific population. In the future, it is necessary to further integrate multi-dimensional information such as individual activity patterns, genetic susceptibility, and environmental triggers to comprehensively analyze the risk contribution of BMI to JIA onset, thereby revealing the association mechanism between the 2 more thoroughly.^[18]^ Future MR studies should also consider a bidirectional approach to examine whether JIA might causally influence BMI. Moreover, performing stratified analyses by JIA subtype, and utilizing larger or more up-to-date JIA GWAS data (for example, from FinnGen) as well as multi-ethnic population data, would be beneficial to validate and extend the current findings. Furthermore, the use of approaches such as radial MR plots or colocalization analysis could help in assessing potential pleiotropy and confirming that the observed association is truly causal.

Studies have shown that baseline obesity in JIA patients is significantly associated with worsening disease activity at the 6-month follow-up, while hip joint involvement is associated with lower BMI levels (suggesting that both obesity and joint involvement may affect disease control and prognosis).^[19]^ Furthermore, a study by Schenck S further pointed out that JIA onset is closely associated with BMI, and its pathogenesis may interact with increased BMI, high-dose glucocorticoid use, functional limitations, and lack of physical activity. Increased BMI not only raises the risk of overweight but may also affect the disease phenotype and long-term prognosis of JIA.^[20]^ In a long-term follow-up cohort of JIA patients in Northern Europe, BMI was significantly associated with disease activity, disability, and health-related quality of life in patients, indicating that BMI is an important factor influencing the clinical outcomes of JIA. A multi-center study in Portugal and Brazil also showed a significant association between BMI and disease activity in JIA patients: as BMI changed, the Juvenile Arthritis Disease Activity Score 27 and inflammatory markers of patients showed an upward trend, which confirms that BMI is an independent risk factor for increased disease activity.^[21]^ Another case–control study found no significant differences in BMI and body composition between JIA patients with well-controlled disease and healthy controls; however, patients with high inflammatory activity exhibited higher BMI, fat mass, and fat mass index, suggesting a potential association between inflammation and fat accumulation.^[22]^

The strengths of this study lie in its 2-sample MR design based on BMI-related SNPs and JIA, which provides stronger evidence for the causal association between the 2. Within the MR framework, the random assignment characteristic of SNPs can minimize biases caused by reverse causality and confounding factors,^[23]^ thereby strengthening causal inference more effectively than observational studies. Methodologically, the study obtained valid data by setting specific screening criteria and used multiple statistical methods (including IVW, WME, MR-Egger regression, and weighted models) to calculate OR, 95% CI, and *P*-values. Meanwhile, Cochran *Q* test was used to assess heterogeneity, Egger-intercept test to detect pleiotropy, and the leave-one-out method to conduct sensitivity analysis (further improving the reliability of the study). Additionally, visualizing data as forest plots, funnel plots, and scatter plots facilitated the intuitive observation of the association between exposure and outcome, data heterogeneity, and consistency in effect directions. These comprehensive methods not only ensure the robustness of the study results but also demonstrate significant advantages in analyzing the epidemiological causal relationship of JIA.

However, this study still has certain limitations. Firstly, the study samples were only obtained from JIA patients of European ancestry, and the applicability of the results to populations of other ethnicities and regions has not been verified. Secondly, as a large-sample statistical study, it did not conduct in-depth stratified analyses of factors such as JIA subtype, disease duration, age at onset, gender, and lifestyle. Thirdly, the study focused on the causal relationship level and did not involve immunological or genetic mechanisms related to JIA onset, which may lead to certain biases. Fourthly, the statistical power of our MR analysis to detect such a tiny causal effect was very low. Using the mRnd tool, we estimated that for an OR of ~1.00175 per 1 SD increase in BMI (~4.5 kg/m^2^), given a BMI sample size of 99,998, a JIA sample size of 15,872, and 35 genetic instruments (total variance explained *R*^2^ ≈ 0.038), the study’s power was only on the order of ~5%, far below the conventional 80% threshold required to reliably detect an effect.

In conclusion, our study findings demonstrate that an increase in BMI exerts only a negligible effect on the risk of onset and disease activity of JIA, and the clinical significance of this association is relatively limited. This also emphasizes that in the long-term management of JIA, attention should be paid to weight management, and measures such as a reasonable diet, moderate physical activity, and health education should be implemented to improve disease control and prognosis.

## Acknowledgments

We would like to express our gratitude to all the databases utilized in this study, as well as to all participants whose data contributed to these databases and thereby supported the conduct of our research.

## Author contributions

**Conceptualization:** Renkun Huang, Jiehua Lu.

**Data curation:** Guohua Jiang.

**Funding acquisition:** Chaoquan Yang.

**Investigation:** Guohua Jiang, Yuchang Gui, Bingtao He.

**Methodology:** Yuchang Gui.

**Project administration:** Renkun Huang, Jiehua Lu.

**Software:** Bingtao He.

**Supervision:** Jianwen Xu.

**Validation:** Yuchang Gui.

**Writing – original draft:** Renkun Huang, Guohua Jiang, Jiehua Lu.

**Writing – review & editing:** Jianwen Xu.
